# Supplementary material for: Male Predominance of Gastric Cancer among Patients with Hypothyroidism from a Defined Geographic Area
Source: J Clin Med. 2020 Jan 3;9(1):135. doi: 10.3390/jcm9010135 (PMC7019714; doi:10.3390/jcm9010135)
Supplement: Supplementary file 1 [file jcm-09-00135-s001.pdf]

Supplemental Table. Patients with active chronic gastritis according to *Helicobacter pylori* status and diagnostic tests.

Study population	***Patients no.***
Patients with active - chronic gastritis	2084
Patients with active - chronic gastritis and negative for *H. pylori* at histology	125
H. pylori infection confirmed by 13C-UBT	78
H. pylori infection confirmed by stool antigen test	47
